# Emodin Attenuated the Kidney Damage of High-Fat-Diet Mice via the Upregulation of Glucagon-Like Peptide-1 Receptor

**DOI:** 10.1155/2021/6662704

**Published:** 2021-05-31

**Authors:** Jinlei Liu, Yao Sun, Hongwei Zheng, Jing Wang, Lili Liu, Bing Song, Haoqiang Zhang

**Affiliations:** ^1^First Affiliated Hospital of Jinzhou Medical University, Jinzhou, China; ^2^Department of Pharmacy, Taikang Xianlin Drum Tower Hospital, Medical School of Nanjing University, Nanjing, China; ^3^Department of Endocrinology, Affiliated Zhongda Hospital of Southeast University, Nanjing, China

## Abstract

**Objective:**

Secretion of glucagon-like peptide 1 (GLP-1) and its effect on target organs were impaired in individuals with obesity. However, its mechanism needs to be further studied. We aim to explore the roles of the receptor of GLP-1 (GLP-1R) involved in high-fat-diet- (HFD-) induced kidney damage improved by emodin.

**Methods:**

Male C57bl/6 mice were fed with HFD diet and therapied by emodin. NRK-52E cells were cultured and treated with palmitic acid or low-density lipoprotein cholesterol (LDL-C). Emodin was used to remedy the NRK-52E cell damage. GW9662 was administrated to block the function of peroxisome proliferator-activated receptor *γ* (PPAR-*γ*). GLP-1 in the plasma was measured by ELISA. PPAR-*γ* and GLP-1R in the kidney and NRK-52E cells were detected by western blotting. The interaction between PPAR-*γ* protein and GLP-1R promoter regions was observed by chromatin immunoprecipitation (ChIP).

**Results:**

Postprandial GLP-1 levels in plasma, as well as PPAR-*γ* and GLP-1R, decreased in kidney tissue of HFD mice, while they were reserved by emodin treatment. Although PPAR-*γ* and GLP-1R were not downregulated by LDL-C, they were suppressed by palmitic acid. Interestingly, GLP-1R mRNA was detected by PCR in the mixture pulled down with PPAR-*γ* antibody. Additionally, downregulation of PPAR-*γ* and GLP-1R by palmitic acid was remanded by emodin. Moreover, GW9662, an inhibitor of PPAR-*γ*, abolished the protective effect of emodin.

**Conclusion:**

The kidney damage of HFD mice seems to be alleviated by emodin via the upregulation of GLP-1R in kidney tissue.

## 1. Introduction

In recent years, the prevalence of obesity [1–3] and diabetes [4, 5] is increasing. Their damages of target organs (including kidney) have become urgent problems. Although diabetic nephropathy is one of the most important causes of end-stage renal disease [6, 7], relatively few studies have been done on obesity-related glomerulopathy for its insidious development [8].

A few studies suggested that obesity damages the kidney of animals and human beings and even results in end-stage kidney disease. However, its mechanism remains unclear [9]. Previous studies have found that the kidney is also rich insulin receptor [10] except the typical insulin-targeted organs: fat, muscle, and liver. Recent work found that insulin resistance is involved in kidney injury in mice induced by HFD [11].

Emodin is a kind of anthraquinone with biological activity extracted from the roots and stems of rhubarb and other Chinese herbal medicines. It has antibacterial, anti-inflammatory, antiulcer, antitumor, regulatory immunity, and antidiabetes effects [12]. Our previous study found that emodin can alleviate insulin resistance in KKAy mice with diabetes [13]. The mechanisms of emodin in diabetic nephropathy have been widely studied. In addition, it has been found that emodin can improve insulin resistance of diabetic mice through PPAR-*γ* [14, 15]. Moreover, PPAR-*γ* is one of the most important targets [16] involved not only in diabetic nephropathy [17] but also in obesity-related glomerulopathy [15]. However, the protective effect of emodin activated PPAR-*γ* involved in obesity-related glomerulopathy still needs to be further studied.

GLP-1 is a polypeptide secreted by L-type cells of the intestinal epithelium, which can stimulate insulin secretion and inhibit glucagon secretion. Therefore, it plays an important role in blood glucose homeostasis [18]. GLP-1 works by binding to GLP-1R on target organs [19, 20]. It has been found that the expression of GLP-1R decreased in the kidney of HFD mice [21]. Therefore, we guess that the dysfunction of GLP-1R signaling pathway may be one of the reasons of kidney damage in obese mice induced by HFD. Although PPAR-*γ* and GLP-1R are both involved in renal injury of obese or diabetic animal models [22–25], the relationship between PPAR-*γ* and GLP-1R is unknown. In other words, as a nuclear receptor, the regulation effect of PPAR-*γ* on the expression of GLP-1R needs to be further explored. Additionally, emodin may regulate GLP-1R via PPAR-*γ*.

To confirm our hypothesis, we fed mice with HFD, and then, emodin treatment was given to detect the regulatory effect of emodin on GLP-1R and PPAR-*γ* in the kidney of mice with HFD. Moreover, NRK-52E cells were cultured and stimulated by LDL-C or palmitic acid and then remedied with emodin. Finally, GW9662 administration and ChIP assays were performed to confirm the role of PPAR-*γ* involved in GLP-1R expression.

## 2. Methods

### 2.1. Experimental Animal Housing and Treatment

**Figure 1:**
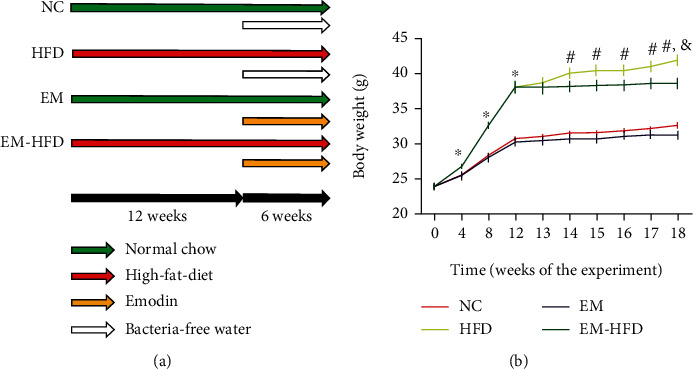
Treatment and body weight changes. (a) Diets and treatments of mice at different time points. (b) “^∗^” shows different body weights of mice with HFD vs. those with NC before emodin was treated, *p* < 0.05; “#” shows different body weights of mice with HFD and emodin vs. those with HFD but without emodin, *p* < 0.05; “&” shows different body weights of mice with NC and emodin vs. those with HFD but without emodin, *p* < 0.05. *n* = 8 in each group.

Healthy C57BL/J mice (male, age 7 weeks, *n* = 40) were purchased from HFK Bioscience Co., Ltd. (Beijing, China). All mice were housed in the specified pathogens free for 1 week before experiments. Animals were randomly divided into 4 groups according to their diets and treatments: normal chow group (NC, *n* = 10), HFD group (HFD, *n* = 10), emodin treatment group (EM, *n* = 10), and HFD group with emodin (EM-HFD, *n* = 10). 2 animals in the HFD group and 1 in the HFD-EM group were detected with hyperglycemia. Additionally, 1 mouse in the HFD-EM group died in the intragastric administration. So, we excluded 4 mice in the NC group (2 mice) and EM group (2 mice). After 12 weeks of feeding with normal chow or HFD diet, mice were administrated with emodin (in DMSO) (50 mg/kg) [13] (cat no. 518-82-1; Solarbio Science & Technology Co., Ltd., Beijing, China) or bacteria-free water of the same frequency and the same volume every other day for 6 weeks (Figure 1(a)). Body weights were measured weekly. All experiments were performed according to the guidance of the Ethics Committee for Experimental Research from the First Affiliated Hospital of Jinzhou Medical University.

### 2.2. Assays on Blood and Urine

At the end of the experiment, mice were sacrificed by cervical dislocation. Blood samples were collected to measure the levels of fasting plasma glucose (FPG), fasting serum insulin (FSI), serum creatinine (Scr), blood urea nitrogen (BUN), total cholesterol (TC), low-density lipoprotein cholesterol (LDL-C), and free fatty acids (FFA).

### 2.3. Measurement of Serum GLP-1

After 8 hours of fasting and 30 minutes of glucose gavage (2 g/kg), blood samples were taken from the heart, centrifuged at 1200 rpm (4°C) for 5 min. After the supernatant was obtained, the level of GLP-1 in serum was detected by an ELISA kit (intraassay precisions CV% < 8% and CV% < 10%) form CUSABIO Technology LLC (CUSABIO, Wuhan, China, Catalogue No.: CSB-E08118m).

### 2.4. NRK-52E Cell Culture and Treatment

The NRK-52E cells were obtained from American Type Culture Collection (Manassas, VA, USA). Cells were cultured in Dulbecco's modified Eagle's medium F-12 supplemented with 10% fetal bovine serum, penicillin (100 U/ml), and streptomycin (100 *μ*g/ml) in an incubator at 37°C with 5% CO2. NRK-52E cells were treated with LDL-C (100 or 200 *μ*g/ml) [26] or palmitic acid (150 *μ*M) [27] for 24 h. And then, NRK-52E cells with 150 *μ*M palmitic acid were treated with emodin (50 *μ*M) [12, 28] and GW9662 (25 *μ*M) [29] at the time point of palmitic acid treatment.

### 2.5. Masson Staining

Tissues from the upper pole of the kidney were collected from freshly sacrificed mice and fixed with 4% paraformaldehyde for 72 h before paraffin sections (5 *μ*m) were obtained. Masson staining was conducted according to the manufacturer's protocol (Wanlei Biotechnology Co. Ltd, Shenyang, China, Catalogue No.: WLA045) similar to our previous study [30].

### 2.6. Western Blotting

Western blotting was carried out according to our previously described protocol [31] and described briefly as follows. Kidney tissue or NRK-52E cells were extracted by radioimmunoprecipitation (RIPA) (Wanlei Biotechnology Co. Ltd, Shenyang, China, Catalogue No.: WLA016a) and measured by a BCA assay according to the manufacturer's instructions (Wanlei Biotechnology Co. Ltd, Shenyang, China, Catalogue No.: WLA004b). Proteins were separated in SDS-PAGE gels and transferred to polyvinylidene fluoride (PVDF) membranes. Rabbit-anti-mouse primary antibodies were used to bind target proteins including PPAR-*γ* (Santa Cruz Biotechnology, Inc., Dallas, Texas, USA, Catalogue No.: sc-390740), GLP-1R (Bioss, Beijing, China, Catalogue No.: bs-1559R), and *β*-actin (Wanlei biotechnology co. Ltd, Shenyang, China, Catalogue No.: WL01845) at 4°C overnight. And then, goat-anti-rabbit secondary antibody conjugated with HRP (Wanlei Biotechnology Co. Ltd, Shenyang, China, Catalogue No.: WLA023a) incubation was performed for binding primary antibodies. The ECL kit (Wanlei Biotechnology Co. Ltd, Shenyang, China, Catalogue No.: WLA006a) was utilized before exposure to detect the protein levels.

### 2.7. ChIP

The ChIP assay was conducted according to the protocol from the manufacturer (Wanlei Biotechnology Co. Ltd, Shenyang, China, Catalogue No.: WLA122). NRK-52E cells were cross-linked by 1% formaldehyde for 10 min at room temperature. After ultrasonic splintering, chromatin solutions were incubated with 4 *μ*g of anti-PPAR-*γ* antibody or with IgG and rotated overnight at 4°C. Complexes were collected with protein A Sepharose beads for 1 h at 4°C. To purify the immunoprecipitated DNA, beads were treated with DNase-free RNase A and proteinase K. And then, DNA was resuspended in distilled water. To amplify the GLP-1R promoter regions containing PPAR-*γ*, 5′-CAAGTCCACGCTGACACTC-3′ and 5′-GCTCTGTAAACAGCTTGATGAA-3′ were used as forward and reverse primers, respectively [32]. After amplification, PCR products were analyzed on a 2% agarose gel. For quantification of the ChIP assay, input genomic DNA and immunoprecipitated DNA were amplified by real-time PCR.

### 2.8. Statistical Analysis

All data were described as mean ± standard deviation. Statistical differences were determined by using Student's *t*-test and one-way ANOVA followed by LSD for multiple comparison test. Data were analysis by SPSS 22.0 (SPSS Inc., Chicago, IL, USA). *p* < 0.05 was considered a significant difference.

## 3. Results

### 3.1. Effect of Emodin on Body Weights

To explore the effect of emodin on body weight of mice with HFD, all mice (8 weeks old) were fed with common chow or HFD for 12 weeks. And then, part mice with common chow or HFD were treated with emodin for 6 weeks (Figure 1(a)). Compared to mice with common chow, body weights of mice increased 24.89% after 12 weeks feeding with HFD. At that time, emodin was used to treat HFD-induced mice with obesity. Interestingly, compared to mice without emodin, emodin prevented body weights increasing from HFD by 8.70% after 6 weeks of emodin treatment (Figure 1(b)).

### 3.2. Effect of Emodin on Biochemical Indexes of Blood and Urine

**Figure 2:**
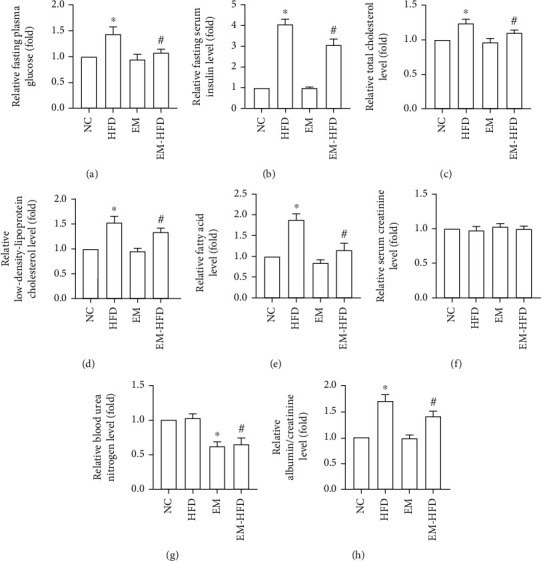
Indexes of glucose or lipid metabolism and renal function of mice. (a–h) “^∗^” shows indexes of the HFD group or EM group vs. the NC group, *p* < 0.05; “#” shows indexes of EM-HFD group vs. HFD group, *p* < 0.05. *n* = 8 in each group.

In order to detect the effect of emodin on homeostasis of glucose and lipid metabolism, FPG, FSI, TC, LDL-C, and FFA were measured. Amazingly, emodin not only decreased the levels of FPG and FSI increased by HFD but also decreased the levels of TC, LDL-C, and FFA elevated in HFD feeding mice (Figures 2(a)–2(e)). For the best exploration of kidney damage alleviated by emodin, Scr, BUN, and urine albumin/creatinine were measured. Although Scr and BUN levels were not increased by HFD, BUN levels were decreased by emodin in mice with or without HFD (Figures 2(f) and 2(g)). Moreover, HFD elevated the levels of albumin/creatinine, while they were decreased by emodin (Figure 2(h)).

### 3.3. Effect of Emodin on GLP-1 in Serum and Pathology in the Kidney

**Figure 3:**
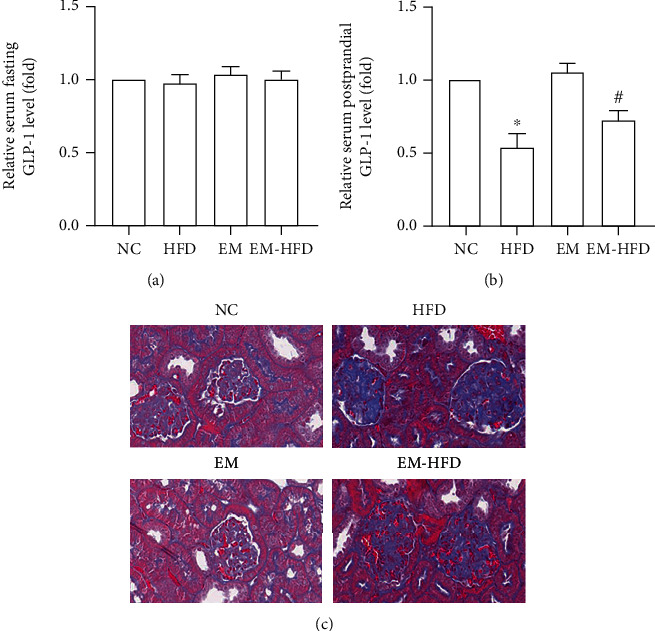
GLP-1 in serum and pathology in the kidney of mice. Results in (a) show no difference of GLP-1 levels among the NC group, HFD group, EM group, and EM-HFD group. (b) “^∗^” shows decreased postprandial GLP-1 levels in HFD mice vs. mice of NC group, *p* < 0.05; “#” shows increased postprandial GLP-1 levels in mice of EM-HFD group vs. mice of HFD group, *p* < 0.05. Results in (c) show more collagen deposition in HFD mice, compared with mice with normal chow and less collagen deposition in EM-HFD mice compared with HFD mice. *n* = 8 in each group.

Although no significant change of fasting GLP-1 was detected in mice with or with HFD (Figure 3(a)), compared with mice administrated with sterile water, the levels of GLP-1 in mice with common chow increased more than 2-folds after glucose intragastric administration for 30 minutes. Additionally, GLP-1 levels after glucose intragastric administration in mice with common chow are lower than those with HFD. However, they were improved by emodin administration (Figure 3(b)). In Masson stain of renal tissue, more collagen fibers were deposited in the glomeruli of mice fed with HFD. Interestingly, the deposited collagen was decreased by emodin (Figure 3(c)).

### 3.4. Effects of Emodin on GLP-1R and PPAR-*γ* in Renal of Mice with HFD

**Figure 4:**
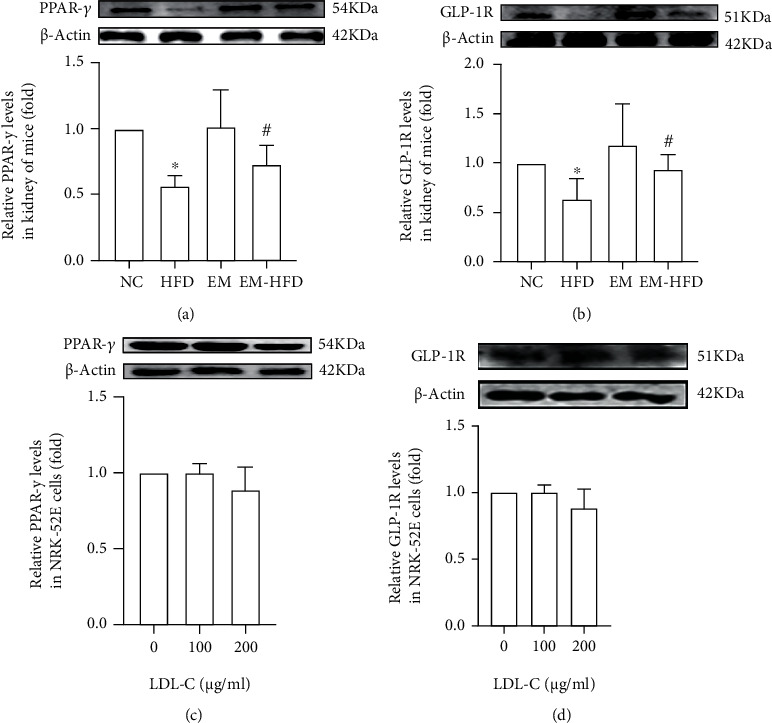
GLP-1R and PPAR-*γ* in the kidney of mice and NRK-52E cells with or without LDL-C. (a, b) “^∗^” shows decreased PPAR-*γ* and GLP-1R levels in HFD mice, compared with NC mice, *p* < 0.05; “#” shows increased PPAR-*γ* and GLP-1R levels in EM-HFD mice, compared with HFD mice, *p* < 0.05. Results in (c, d) did not show different PPAR-*γ* or GLP-1R levels among NRK-52E cells with different concentrations of LDL-C. All results were repeated at least 3 times.

Owing to the levels of GLP-1 levels after administrated with glucose were decreased by HFD and increased by emodin, GLP-1R levels of mice with or without HFD (emodin) were measured. Kidney tissue of mice with HFD showed downregulated GLP-1R levels, compared with those of mice with common chow. Moreover, GLP-1R levels were upregulated by emodin (Figure 4(b)). In addition, PPAR-*γ*, one of the most important targets of emodin, was detected. Undoubtedly, PPAR-*γ* levels decreased in the kidney of mice with HFD and increased by emodin (Figure 4(a)).

### 3.5. LDL-C Did Not Influence the Levels of GLP-1R or PPAR-*γ* in NRK-52E Cells

To further explore the mechanism of kidney damages, especially for the damage of renal tubules, NRK-52E cells were cultured and treated with LDL-C or palmitic acid. Although LDL-C levels are significantly increased in mice with HFD, in this present study, LDL-C treatment did not change the levels of GLP-1R or PPAR-*γ* in NRK-52E cells at the concentration of 100 *μ*g/ml or 200 *μ*g/ml (Figures 4(c) and 4(d)).

### 3.6. Effects on GLP-1R or PPAR-*γ* in NRK-52E Cells of Palmitic Acid and Emodin

**Figure 5:**
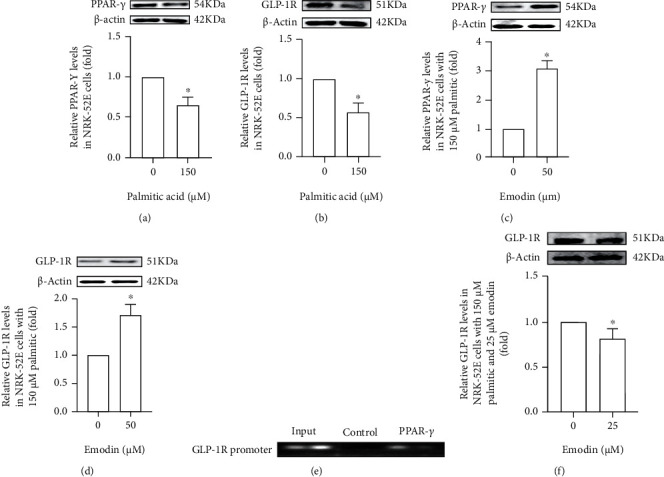
GLP-1R and PPAR-*γ* in NRK-52E cells with palmitic acid. (a, b) “^∗^” shows decreased PPAR-*γ* and GLP-1R in NRK-52E cells with 100 *μ*M palmitic acid, compared to those without palmitic acid. (c, d) “^∗^” shows increased PPAR-*γ* and GLP-1R in NRK-52E cells with 100 *μ*M palmitic acid and 50 *μ*M emodin, compared to those with 100 *μ*M palmitic acid but without emodin; Results in the figure show the interaction between PPAR-*γ* and GLP-1R promoter. (f) “^∗^” shows decreased GLP-1R in NRK-52E cells with 100 *μ*M palmitic acid, 50 *μ*M emodin, and 25 *μ*M GW9662, compared to those with 100 *μ*M palmitic acid and 50 *μ*M emodin, but without GW9662. All results were repeated at least 3 times.

Although LDL-C did not suppress the levels of GLP-1R or PPAR-*γ* in NRK-52E cells, palmitic acid significantly downregulated the levels of GLP-1R or PPAR-*γ* in NRK-52E cells at the concentration of 150 *μ*M (Figures 5(a) and 5(b)). However, the downregulation of GLP-1R or PPAR-*γ* was restored by emodin at the concentration of 50 *μ*M (Figures 5(c) and 5(d)).

### 3.7. Interactions between PPAR-*γ* and GLP-1R

The expression of GLP-1R needs the transcription and translation of GLP-1R gene. To uncover the interaction between PPAR-*γ* and GLP-1R, ChIP assay was carried out. GLP-1R promoter sequence was observed by real-time PCR in the immunoprecipitated DNA (Figure 5(e)). To further explore the regulation of PPAR-*γ* to GLP-1R, GW9662, a specific PPAR-*γ* inhibitor, was used to treat NRK-52E cells with palmitic acid and emodin. Interestingly, elevated GLP-1R levels were suppressed by GW9662 (Figure 5(f)).

## 4. Discussion

The renal damage is one of the most important target organ injury in obesity individuals [8, 33–36] for the prevalence of obese patients [3]. Although it could even result in end-stage kidney diseases and contribute to kidney failure [37], only a few attentions were drawn from researchers and patients for its hidden process. Here, not only impaired glucose metabolism but also increased urine albumin/creatinine was measured in mice with HFD. GLP-1 level in the serum after glucose intragastric administration was decreased in HFD feeding mice. It showed that systemic GLP-1 secretions were impaired in mice with HFD. Similar findings insisted that GLP-1 analog prevents obesity-related glomerulopathy by inhibiting excessive autophagy [24]. Mice with HFD exhibited downregulated GLP-1R in the kidney. This is consistent with previous research [21]. Additionally, liraglutide, a kind of GLP-1 analog, improved the outcomes of diabetic nephropathy with similar mechanism of obesity-related glomerulopathy.

Downregulated PPAR-*γ* levels in renal tissue were measured in this present study. Owning to emodin is one of the ligands of PPAR-*γ* [38] and showed antidiabetic nephropathy effect in previous studies [39, 40]. Additionally, PPAR signaling pathway is involved in the process of obesity-related glomerulopathy [11]. So, we hypothesized that emodin may exhibit protective effect on obesity-related glomerulopathy. As far as we know, there is no report about emodin and obesity-related glomerulopathy. Therefore, emodin was used to remedy HFD-induced kidney damage. Interestingly, despite upregulated urine albumin/creatinine, downregulated GLP-1R by HFD was restored by emodin administration. Although the relationship between GLP-1R and urine albumin/creatinine is uncovered, we focus on the regulatory mechanism of GLP-1R in this present study.

In order to investigate the regulatory mechanism of GLP-1R expression, LDL-C and palmitic acid were used to induce damage of NRK-52E cells. Although 100 *μ*g/ml LDL-C is enough to affect the proliferation of renal tubular epithelial cells [26], in this research, 100 *μ*g/ml or 200 *μ*g/ml LDL-C failed to induce the downregulation of PPAR-*γ* and GLP-1R. This may account for oxidized cholesterol but not native cholesterol involved in the damage of renal tubular epithelial cells [41]. However, palmitic acid significantly regulated the decrement of GLP-1R and PPAR-*γ* of NRK-52E cells.

To clarify the effect of emodin on GLP-1R, emodin was used to treat NRK-52E cells with palmitic acid. Interestingly, decreased PPAR-*γ* and GLP-1R levels were reversed by emodin. GLP-1R is a kind of G protein-coupled receptor, which works by binding to ligands [42]. Additionally, its downstream second messenger signaling pathway is depending on the expression of GLP-1R. GLP-1R promoter was detected in the mixture pulled down by PPAR-*γ* primary antibody in a ChIP assay. It suggested the exit of interaction between PPAR-*γ* and promoter sequence of GLP-1R. In another word, PPAR-*γ* may influence the expression of GLP-1R and the downstream signaling pathway involved in injury of renal tubular epithelial cells. To verify the regulatory effect on GLP-1R expression of PPAR-*γ*, GW9662, a selective PPAR-*γ* inhibitor, was used. Amazingly, GW9662 partly abolished the recovery effect on GLP-R of emodin.

Although we have preliminarily explored the protective effect of emodin on kidney damages from HFD, there are still some limitations. Firstly, as a basic experiment, especially for an experiment that the main protein was measured by a semi-quantitative method, we failed to calculate the power for the sample size scientifically. With such a small sample basic experiment, there is a long way to clinical usage of emodin in clinical work. Secondly, emodin upregulates the levels of GLP-1R and PPAR-*γ* decreased in HFD mice in our experiment. However, emodin also protects individuals from weight gain, hyperglycemia, and hyperlipidemia in this work and others [43, 44]. To exclude these potential mechanisms of beneficial effect from emodin, mice with hyperglycemia were removed from this experiment. In addition, LDL-C was used to treat NRK-52E cells. However, there is still slightly difference of plasma glucose of mice in each group. Moreover, other lipid levels were not considered in this work. Although body weight-matched mice in the HFD and EM-HFD groups may partly avoid the effect of weight loss effect of emodin, enough numbers of body weight-matched mice are hard to obtain. Thirdly, we used mice and rat cell line in vivo and in vitro, respectively, in this present work. The species difference may limit the scientific conclusion of this research. Fourthly, we just discussed the effect on GLP-1R expression regulated by PPAR-*γ* and confirm the impaired GLP-1 in serum and GLP-1R in the kidney but failed to explore the important role of GLP-1R in renal injury of obese individuals in this study. The GLP-1R knockout animal model remains needed to investigate the essential role of GLP-1R in further research.

## 5. Conclusion

In general, we demonstrated that emodin may alleviate the kidney damage induced by HFD via GLP-1R. Additionally, the regulation of GLP-1R may partly depend on the function of PPAR-*γ* activated by emodin.

## Data Availability

All data used to support the findings of this study are available from the corresponding authors upon request.
